# Renal Oncocytoma Treated With Stereotactic Body Radiation Therapy in a Patient With Prior Pancoast Tumor

**DOI:** 10.7759/cureus.78150

**Published:** 2025-01-28

**Authors:** Nathan Gould, Brandon Brower, Mosab Maree, A. Rashid Dar

**Affiliations:** 1 Medicine, Schulich School of Medicine and Dentistry, Western University, London, CAN; 2 Medical Imaging, London Health Sciences Centre, Western University, London, CAN; 3 Radiation Oncology, Schulich School of Medicine and Dentistry, Western University, London, CAN

**Keywords:** cisplatin-based chemotherapy, non-small cell lung carcinoma (nsclc), palliative radiation therapy, pancoast tumour, renal oncocytoma, small renal masses, stereotactic body radiotherapy (sbrt), vinblastine

## Abstract

A renal oncocytoma is a benign tumor of the kidney, typically treated surgically or via ablation therapy, while a Pancoast tumor is a rare tumor of the lung apex, most often non-small cell lung cancer (NSCLC), usually managed with neoadjuvant chemoradiation followed by surgery. In this report, we discuss the diagnosis and management of a 72-year-old man who developed a renal oncocytoma and a recurrent Pancoast tumor.

At the time of our patient's treatment for his renal oncocytoma, thermal and radiofrequency ablation were not frequently employed, and active surveillance was also not the standard of care. Therefore, a partial nephrectomy was offered but declined by the patient over quality of life concerns. Following a discussion with radiation oncology, the patient instead opted for stereotactic body radiation therapy (SBRT), which was well tolerated. All clinical and imaging follow-up revealed excellent control of the lesion with no progression. To our knowledge, this is one of the first reported cases of an oncocytoma successfully treated with SBRT.

The patient’s stage 3 NSCLC Pancoast tumor was treated with the vinblastine cisplatin radiation therapy (VCRT) protocol, first developed at the Verspeeten Family Cancer Centre in London, Ontario. It consisted of four cycles of vinblastine and cisplatin, with the last two cycles administered concurrently with radiation therapy. This treatment produced excellent symptom control and an impressive nine years of disease-free survival. When the Pancoast tumor re-emerged at the end of this interval, it was treated with palliative reirradiation, and symptom control was excellent for another 10 months until a right-sided occipital lobe metastatic lesion was discovered and the patient succumbed to his disease.

This report provides a clinical example of the viability and safety of SBRT for the treatment of renal oncocytoma. Importantly, SBRT has shown efficacy in treating renal cell carcinoma, the malignant differential of a renal oncocytoma. Clinicians should therefore consider SBRT for a renal oncocytoma when patients decline surgery (or ablation therapy) or when the pathologic diagnosis of the lesion is in doubt. Our case also provides an example of the successful treatment of a non-resectable NSCLC Pancoast tumor with the VCRT regimen and successful palliative re-irradiation of a recurrent Pancoast tumor.

## Introduction

Renal oncocytoma

Renal oncocytomas are benign lesions of the kidney, constituting about 5% of renal tumors, and are often discovered incidentally on imaging done for another indication [1]. They typically present as solitary, unilateral lesions on work-up. Despite some distinguishing features (such as a central scar and hypervascularity), no radiologic criteria with which to reliably distinguish oncocytomas from malignant kidney lesions - namely, renal cell carcinoma (RCC) - exists [2]. Therefore, the diagnosis of oncocytoma requires a kidney biopsy and histologic analysis. Characteristic features of an oncocytoma under the microscope include tubular structures and nests lined by cells containing a granular and eosinophilic cytoplasm [3]. Oncocytomas fall under the umbrella of small renal masses (SRMs), defined as renal masses <4 cm, and must therefore be differentiated from other SRMs, especially given the widely varying natural history of such lesions. The differential for an oncocytoma includes RCC, metastatic cancer, angiomyolipoma, and metanephric adenoma, among other entities [3]. The overwhelming majority of oncocytomas are benign and can therefore be cured with surgical excision, which, depending on the size of the lesion, is either a total or partial nephrectomy [4]. Thermal or radiofrequency ablation is another treatment option, although recurrence rates tend to be higher compared to surgery. Nonetheless, ablation can be repeated if necessary, producing similar outcomes to surgery with multiple treatments [5]. Overall, the majority of biopsy-proven oncocytomas are treated surgically [4]. Radiation, in particular stereotactic body radiation therapy (SBRT), is not commonly employed in oncocytoma treatment. Recent studies [6] have compared its efficacy with that of surgery and ablation, but to the best of our knowledge, we present one of the first examples of SBRT used in the clinic to treat an oncocytic lesion of the kidney. Herein, we review our experience with this treatment approach and review the relevant literature.

Pancoast tumor

Tumors of the lung apices that develop above the superior pleuro-pulmonary margin and the first rib are termed “Pancoast tumors.” They comprise approximately 3-5% of lung cancers and over 95% of the time are non-small cell lung cancers (NSCLCs). Because of their proximity to important structures (ribs, sympathetic chain, brachial plexus, and subclavian vessels), Pancoast tumors can often present via two clinical manifestations. The first is Horner’s syndrome, which presents as ipsilateral miosis, partial ptosis, anhidrosis, and facial flushing due to tumor destruction of the stellate ganglion of the sympathetic chain. The second is Pancoast syndrome, which presents as a triad of pain (in the ipsilateral neck, chest wall, shoulder, axilla, and ulnar distribution of the arm/forearm), Horner’s syndrome, and weakness/atrophy of the intrinsic hand muscles due to tumor disruption of the brachial plexus [7]. Because these tumors tend to invade the chest wall structures as opposed to the lung parenchyma, they are less likely to initially present with the typical lung cancer symptoms of dyspnea, coughing, and hemoptysis [8].

Pancoast tumors are easily missed on chest X-rays (CXR) due to their location and shadowing [8]. Rather, imaging of query Pancoast tumors for confirmation of diagnosis, evidence of local progression, and preoperative planning involves CT or PET scans, of which the latter allows for the preoperative staging of lymph nodes [7]. MRI is also useful for better visualization of the subclavian arteries, spine, neural foramina, and brachial plexus [8]. Percutaneous transthoracic needle biopsy is the gold standard for tissue sampling. Due to Pancoast tumors’ invasion of the chest wall, they are automatically a minimum of T3 in TNM staging [7]. The modern standard of care for Pancoast tumors is neoadjuvant chemoradiation followed by surgery [9].

Here we present the successful management of a non-resectable NSCLC Pancoast tumor with the vinblastine cisplatin radiation therapy (VCRT) chemoradiation regimen, which was first developed in London, Ontario. Successful palliative reirradiation of a recurrent Pancoast tumor is also described and discussed.

## Case presentation

A 72-year-old previously healthy Caucasian man with a 25-pack-year smoking history (quit in 2002) initially presented to the emergency department in May 2003 due to a sudden onset of sharp pleuritic pain in his left chest. It also radiated into the left axillary and scapular regions. He had no concurrent shortness of breath (SOB), cough, hemoptysis, or other symptoms of note and reported pain relief from taking six acetylsalicylic acid (ASA) 81 mg tablets per day and raising his arms overhead. His initial workup included a CXR that demonstrated an ill-defined left apical intraparenchymal mass suspicious for a Pancoast tumor (image unavailable). The radiologist at the time recommended following up with a CT thorax to better elucidate the pathology. This CT was completed a month later and, similar to the CXR, revealed a locally extensive, 3.5 x 4.2 cm solitary mass in the apical-posterior segment of the left upper lobe involving the left intervertebral foramen at the level of the T1 vertebral body (image unavailable). The mass was suspicious for a neurogenic neoplasm or (less likely) a Pancoast tumor; however, a subsequent CT-guided fine needle aspiration biopsy would confirm the latter as it found the cells were best characterized as NSCLC, specifically large cell type. The CT findings led to the determination that this was a stage 3 NSCLC Pancoast tumor, T4N0M0. It was staged as T4 due to bone invasion and staged as N0 due to no nodal involvement seen on imaging.

**Figure 1:**
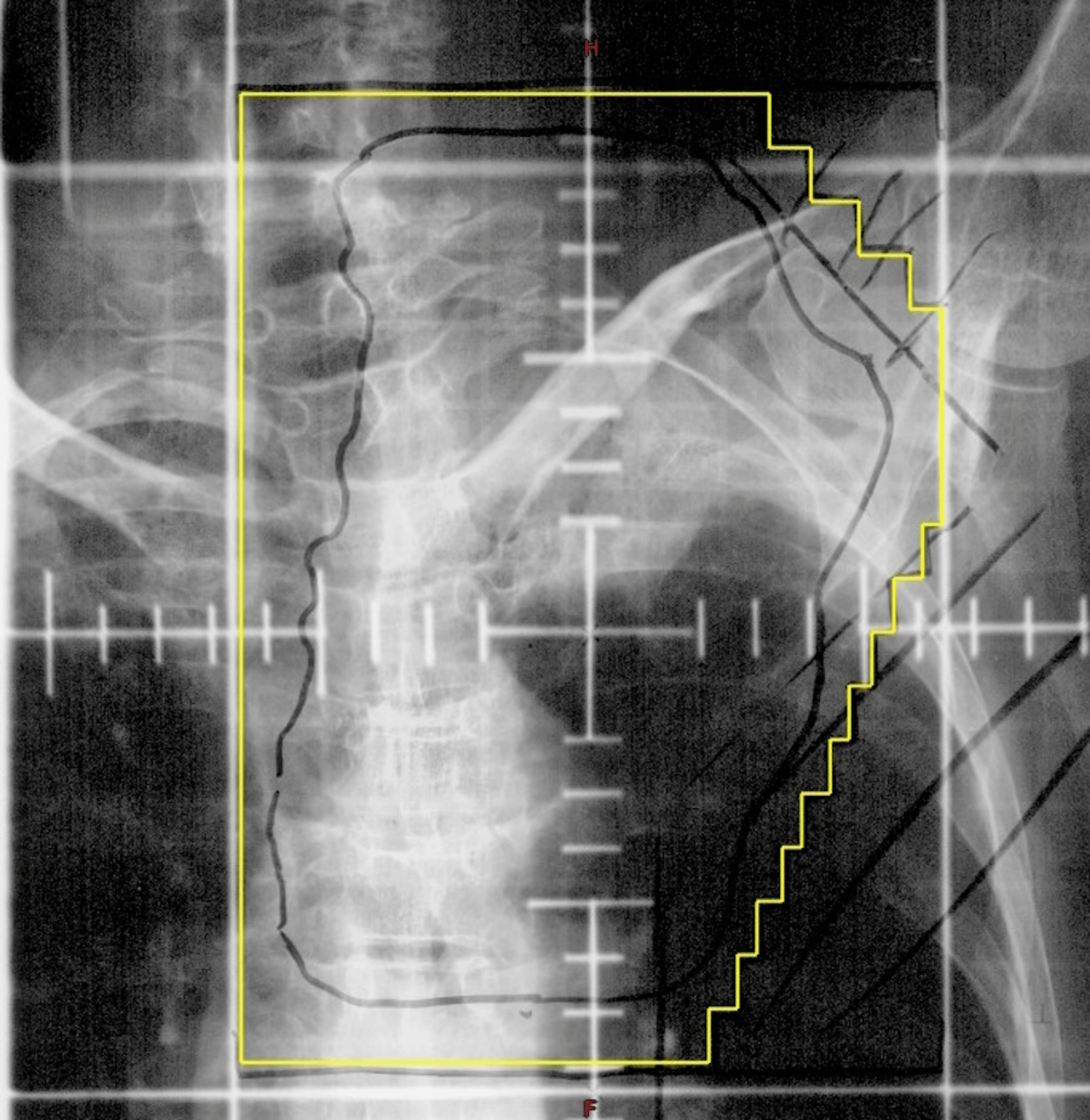
Chest X-ray showing the thoracic radiation plan targeting the left lung apex Posterior-anterior (PA) View

During thoracic multidisciplinary rounds, it was recommended this cancer be treated with the VCRT chemoradiation protocol. This involved two cycles of induction chemotherapy with vinblastine and cisplatin, followed by another two cycles of these chemotherapy agents plus thoracic radiation therapy targeting the left lung apex (Tables 1-2, Figure 1). This treatment commenced in August 2003. The patient developed pancytopenia during his induction chemotherapy treatment, but this eventually resolved, and he had no fevers.

**Table 1:** VCRT chemoradiotherapy schedule Each cycle length is 21 days. BSA: body surface area; IV: intravenous; VCRT: vinblastine cisplatin radiation therapy

Drug	Dose	Days	Cycle
Cisplatin	33 mg/m^2^ BSA IV	1-3	1-4
Vinblastine	2 mg/m^2^ BSA IV	1-3	1-2
Vinblastine	1.4 mg/m^2^ BSA IV	1-3	3-4
Radiation therapy	60 Gy in 30 fractions	Begin day 1 of cycle 3	3-4

**Table 2:** VCRT thoracic radiotherapy plan AP: anterior/posterior; POP: parallel opposed pair

Phase	Technique	Dose	Fractions
1	AP/PA POP	36 Gy	18
2	Oblique POP off cord	14 Gy	7
3	Oblique POP boost	10 Gy	5

In all, this treatment was designed to not exceed spinal cord radiation tolerance, and the patient was advised of this possible risk of radiation. He completed the entirety of this six-week treatment by October 2003 with no significant acute or late toxicities. Early the following year, the patient reported that his anterior chest and arm pain had almost completely disappeared. Around that same time, the prospect of surgery was raised in response to a follow-up CT thorax revealing a residual 2 x 2 cm left apical mass (image unavailable). However, thoracic surgery decided against this given his age and the difficult location of the tumor (involving the first rib). Subsequent regularly scheduled follow-ups for the next two years were essentially normal; some peripheral neuropathy (a typical side effect of vinblastine) following treatment persisted for a year but eventually resolved.

Three years later, in September of 2006, the patient presented to the ER with a week-long hemoptysis of a daily half-cup of dark red blood. He was given antibiotics, and it resolved after a week’s time. It was determined to be caused by radiation therapy-induced telangiectasia, but the recurrence of his lung cancer could not be excluded. Then again, in September of 2008, the patient presented to the ER with another massive hemoptysis (bright red blood filling three cups). Bronchoscopy identified the source of the bleeding as the left upper lobe. Subsequent bronchoscopy, chest X-rays, CTs, and cytology investigations revealed no evidence of tumor recurrence, and thus the hemoptysis was again thought to be due to post-radiation telangiectasia, his being on ASA, and his bearing down with coughing.

**Figure 2:**
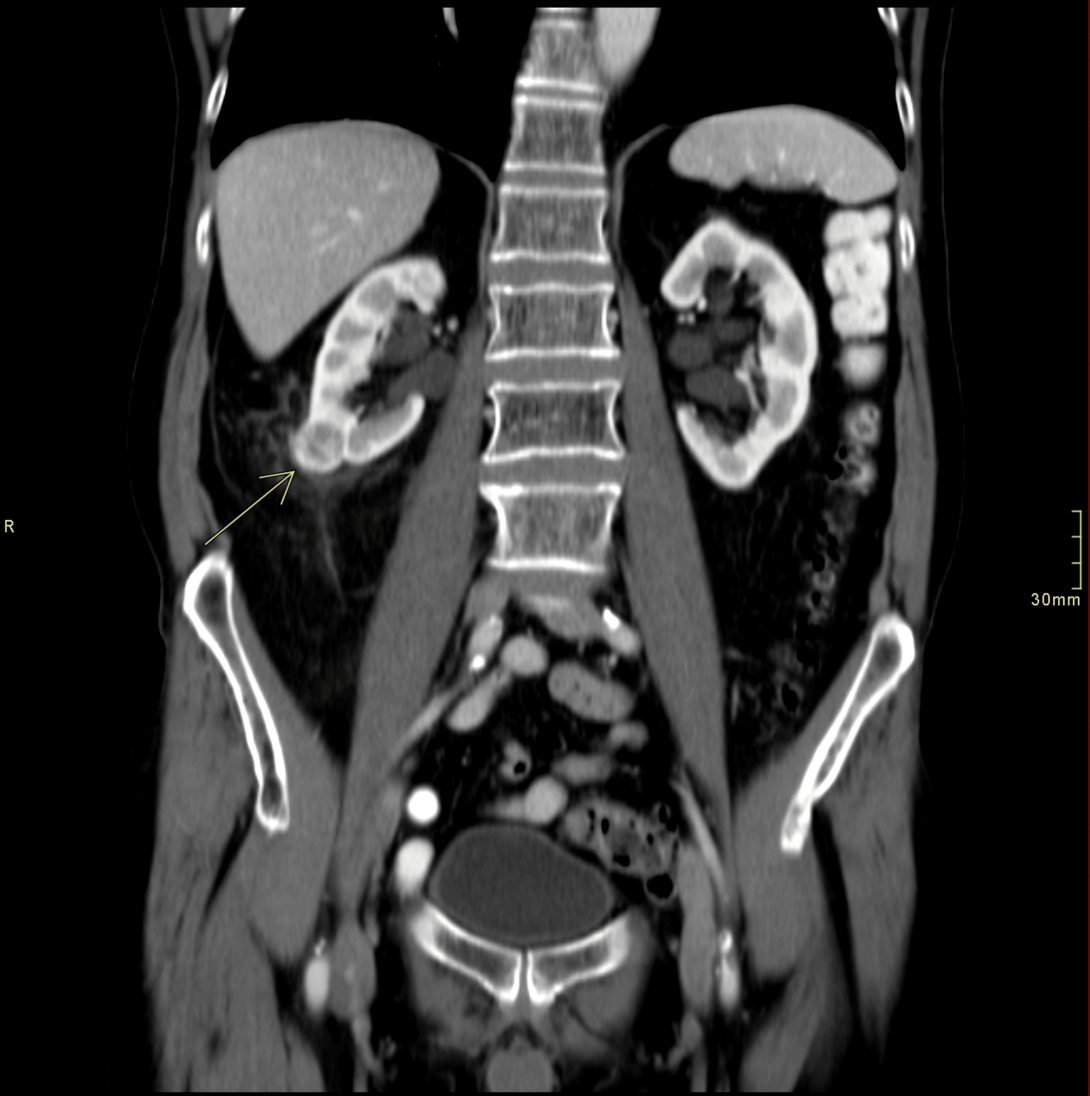
Coronal abdomen and pelvis CT scan with IV contrast/abdomen window showing a 2.0 x 2.3 cm exophytic right lower pole heterogeneously enhancing lesion consistent with an oncocytoma The arrow points to the kidney lesion

**Figure 3:**
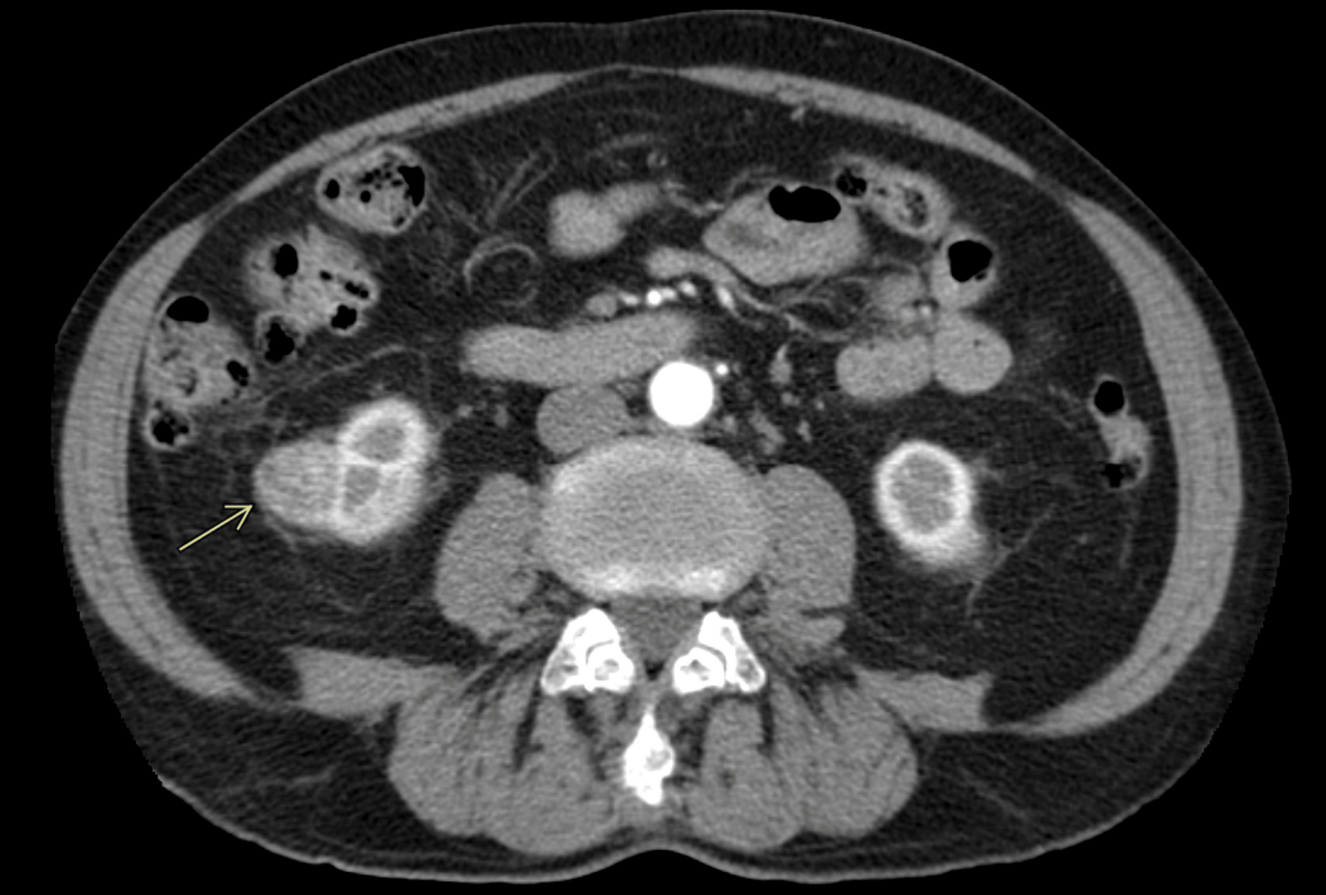
Axial abdomen and pelvis CT scan with IV contrast/abdomen window showing a 2.0 x 2.3 cm exophytic right lower pole heterogeneously enhancing lesion consistent with an oncocytoma The arrow points to the kidney lesion

**Figure 4:**
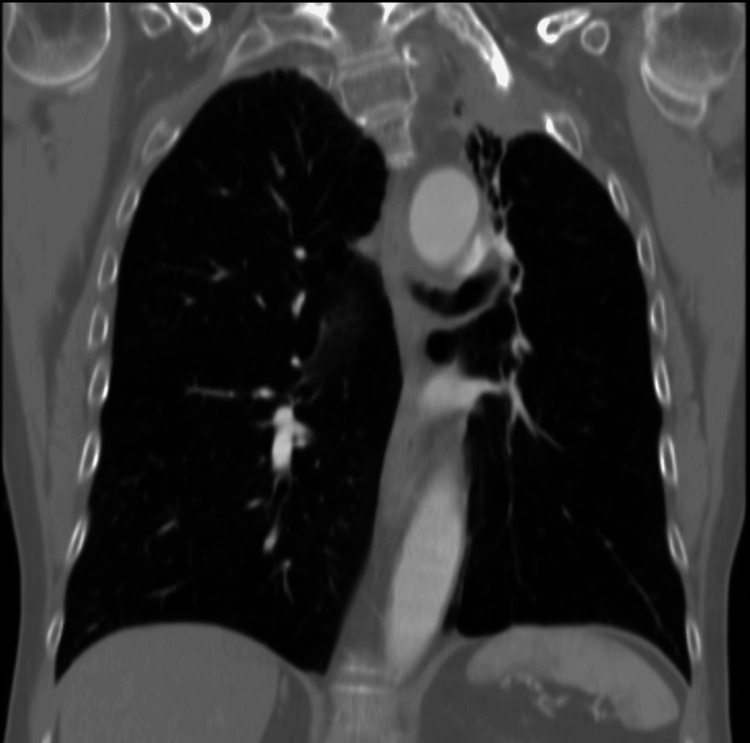
Coronal CT scan with IV contrast of the lungs. There are fibrotic changes, particularly in the left apical lung, with slight thickening of the more central airways on the left

In further work-up of the hemoptysis from over a year prior, a CT scan (because the patient declined bronchoscopy) was performed in March 2010. It revealed a new 2.0 x 2.3 cm exophytic, heterogeneously attenuating cortical renal mass of the right kidney (Figures 2-3) that was not seen on previous CTs (such as CT thorax scans in 2004 and 2007). As well, this scan showed a stable appearance of his lungs compared to previous CTs, with no evidence of Pancoast tumor recurrence (Figure 4).

**Figure 5:**
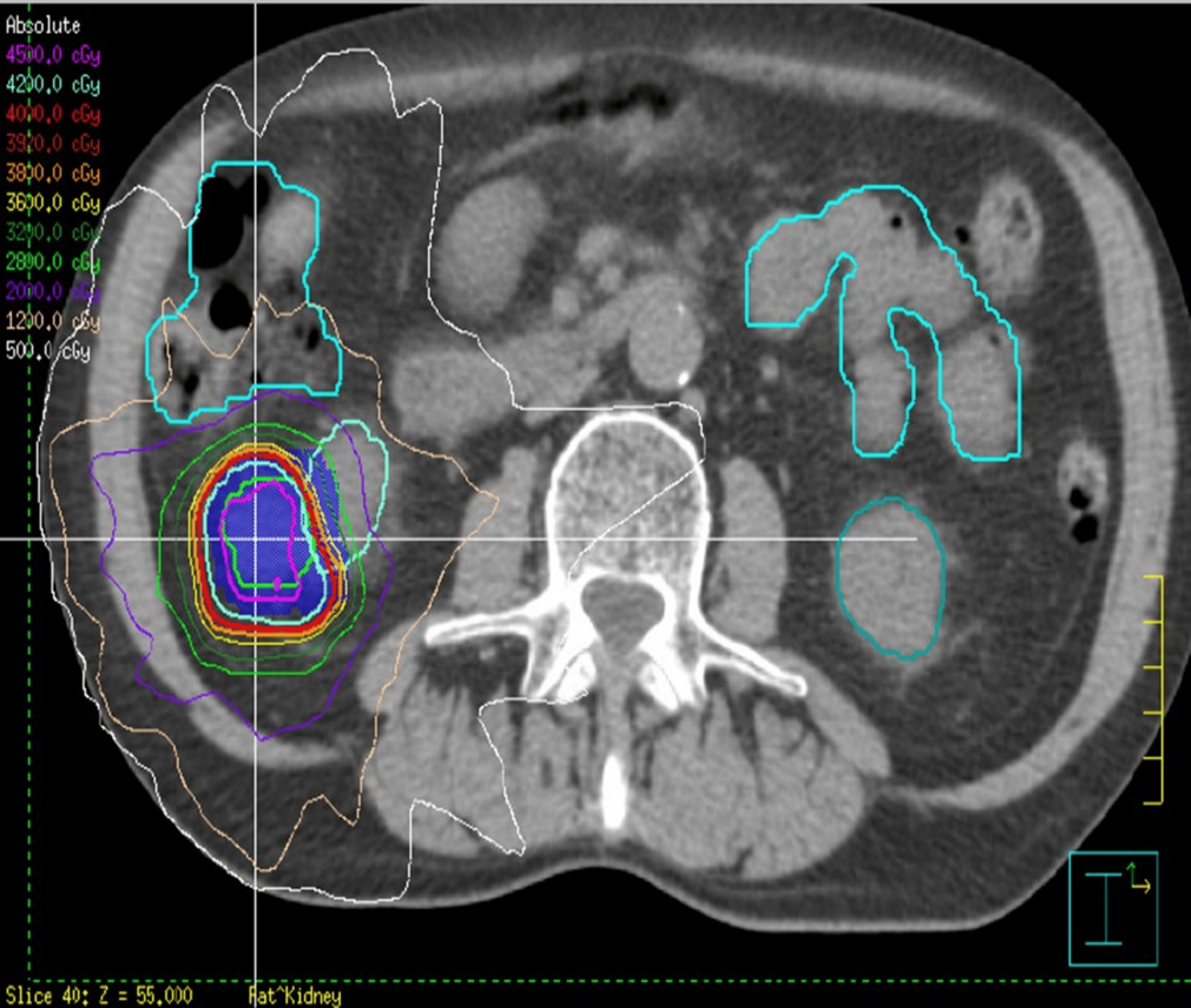
SBRT radiotherapy plan targeting the oncocytoma of the right kidney Axial view. Green contour is GTV. Shaded blue region is PTV. SBRT: stereotactic body radiation therapy; GTV: gross tumor volume; PTV: planning target volume

**Figure 6:**
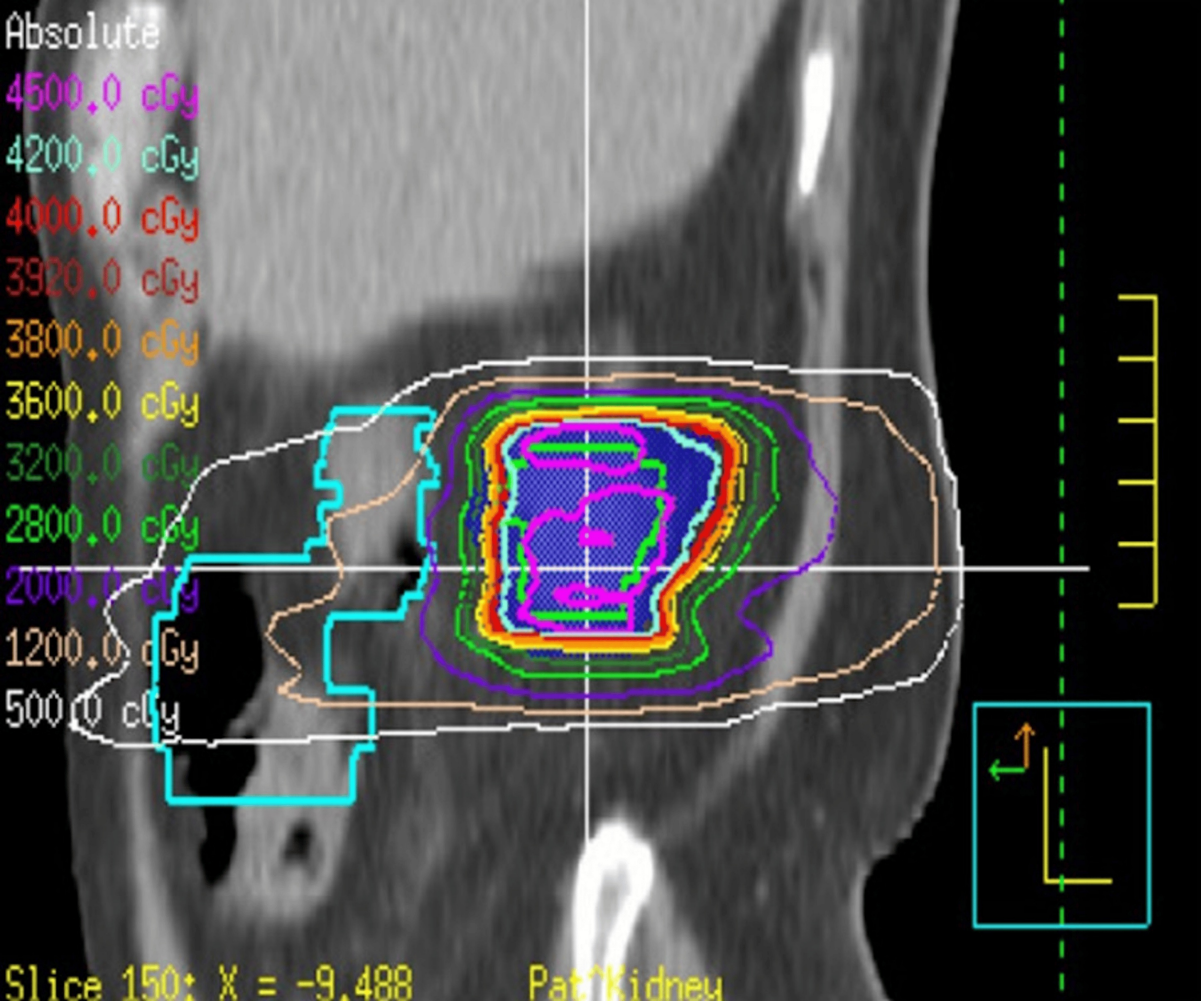
SBRT radiotherapy plan targeting the oncocytoma of the right kidney Sagittal view. Green contour is GTV. Shaded blue region is PTV. SBRT: stereotactic body radiation therapy; GTV: gross tumor volume; PTV: planning target volume

**Figure 7:**
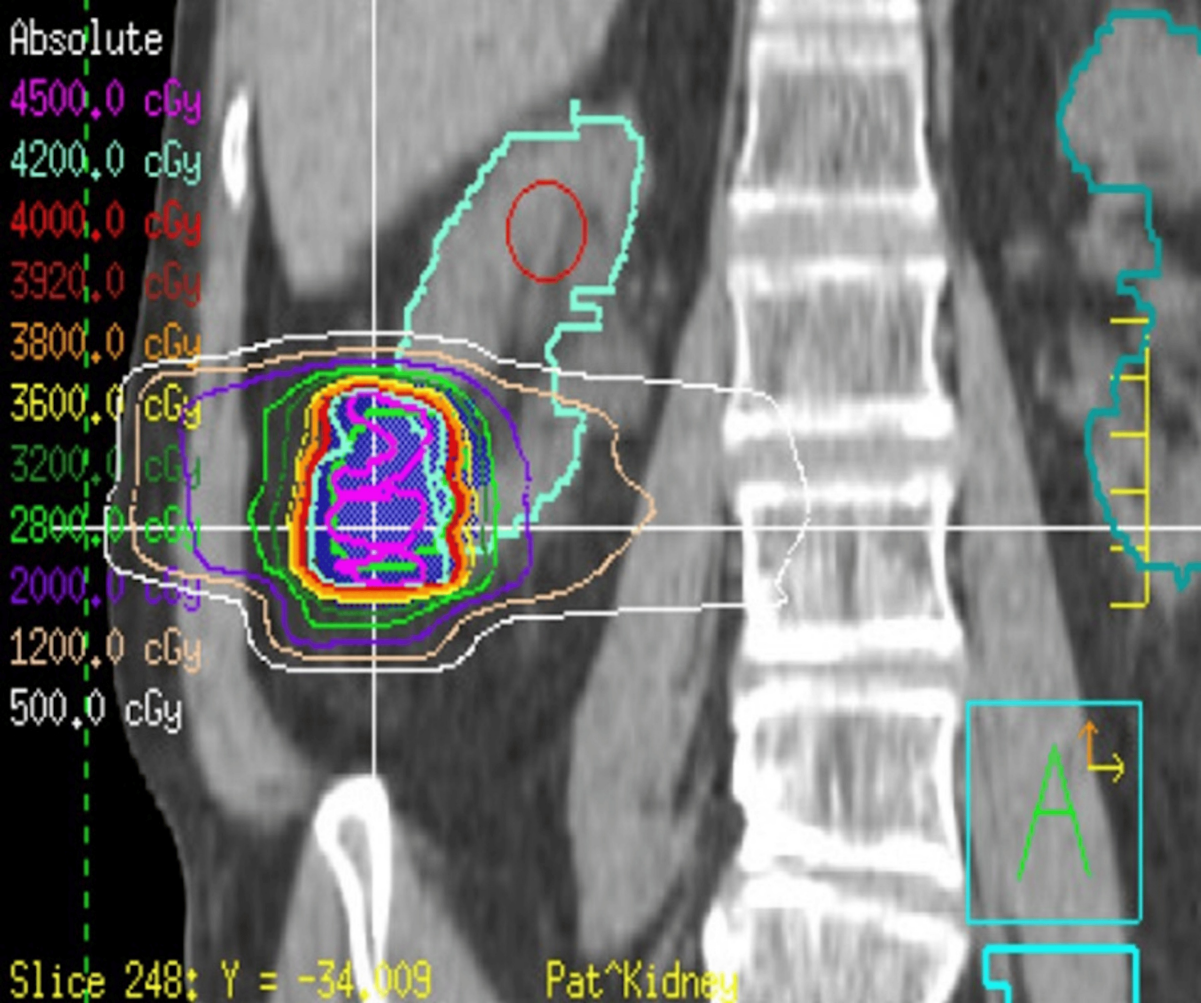
SBRT radiotherapy plan targeting the oncocytoma of the right kidney Anterior-posterior (A/P) view. Green contour is GTV. Shaded blue region is PTV. SBRT: stereotactic body radiation therapy; GTV: gross tumor volume; PTV: planning target volume

Concerned about potential metastasis from the primary lung cancer, an ultrasound-guided biopsy of the right kidney lesion was conducted, the pathology of which revealed cells focally very positive for CK AE1/AE3 but negative for CK 7 and vimentin. The colloidal iron stain was also negative. The pathologist at the time concluded that the features were in favor of an oncocytoma. Nonetheless, a laparoscopic partial nephrectomy for removal of the lesion was advised by urology, as there was a possibility of it being a new primary kidney malignancy or metastasis from the Pancoast tumor. Also, active surveillance of SRMs was not the standard of care at the time, and there was a possibility of the patient becoming symptomatic from the lesion in the future. However, the patient refused surgery due to quality of life concerns and, through discussion with radiation oncology, elected to undergo radiation treatment for his oncocytoma instead. After being informed of the risks and benefits and consenting to this therapy, in June 2010 the patient underwent stereotactic radical radiation therapy of 40 Gy to 90% isodose volume given in four fractions over one week on alternating days (Table 3, Figures 5-7). Standard immobilization was employed using an alpha cradle, and no special motion management was used at the time.

**Table 3:** Radiation dose-volume prescription and dose falloff for various pertinent regions of interest (ROIs) for treatment of the renal oncocytoma GTV: gross tumor volume

Region of interest	Volume (cm³)	Minimum dose (cGy)	Maximum dose (cGy)	Mean dose (cGy)	Standard deviation
Spinal cord	57.8102	3.0	568.3	73.1	123.3
External	17230.7	0.0	4680.5	110.4	389.6
Liver	981.576	0.0	238.9	12.8	13.6
Left kidney	155.401	2.9	279.5	39.0	55.4
Right kidney	104.985	18.9	4677.9	869.8	1184.5
Stomach	415.018	1.0	542.6	32.4	58.2
Small bowel	717.884	1.0	1621.6	73.5	159.9
GTV	16.1376	3089.1	4680.5	4408.5	234.2

This treatment was well tolerated by the patient and was planned such that the surrounding tissues (adrenal glands, bowel, and liver) were kept within normal tissue radiation constraints. He also suffered no side effects, including no urinary symptoms, no pain/cramps, and had normal bowel movements and appetite. All subsequent yearly clinical and CT follow-ups of the oncocytoma showed excellent control of the lesion with some fluctuations in size but no progression. It was considered a stable disease without regression, which was attributed likely both to the low proliferation of the oncocytoma and the possible contribution from the radiotherapy treatment.

**Figure 8:**
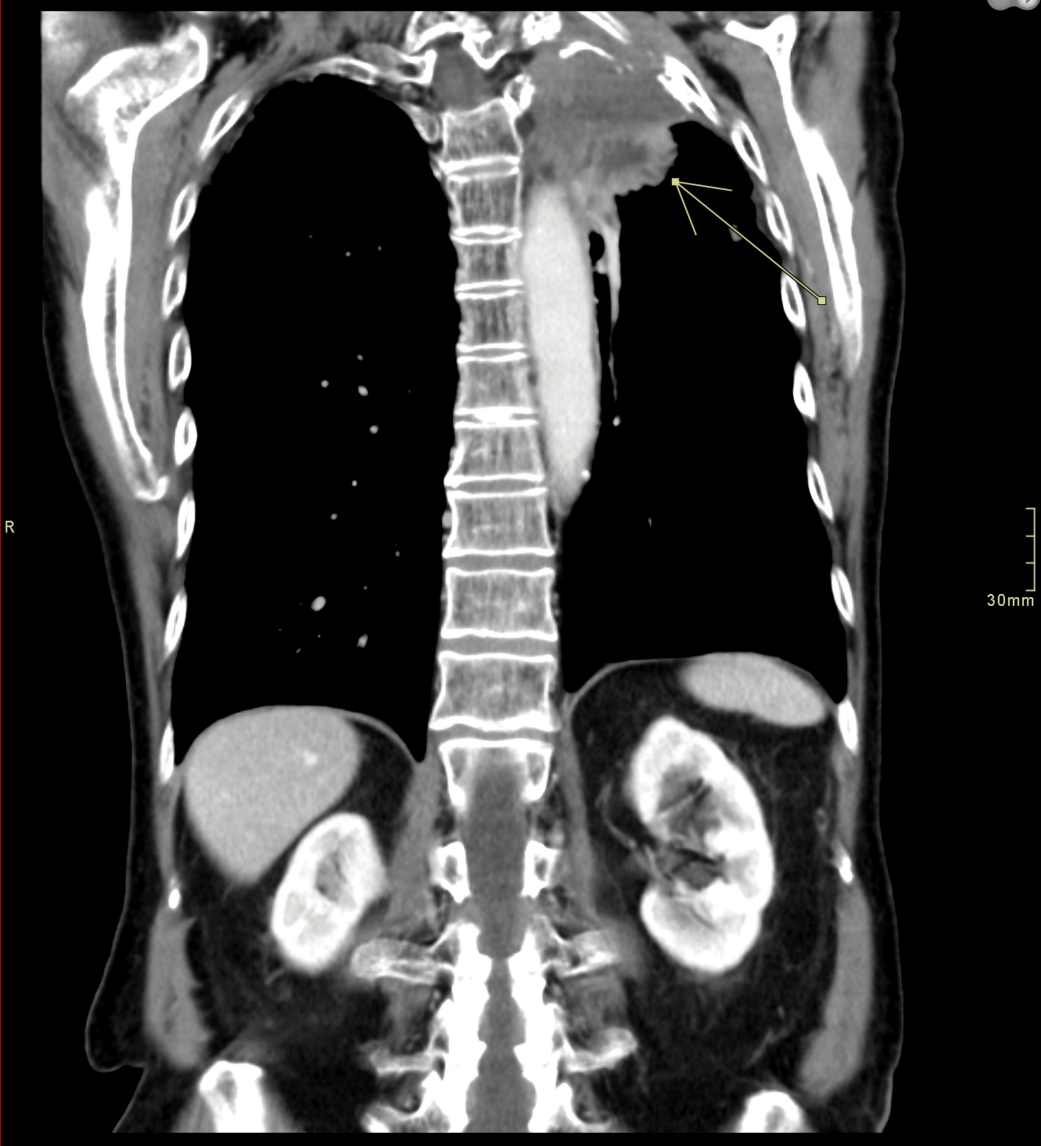
Coronal CT thorax and abdomen/mediastinal window with IV contrast revealing a new mass in the LUL suggesting recurrence of the Pancoast tumor LUL: left upper lobe The arrow points to the lung lesion

**Figure 9:**
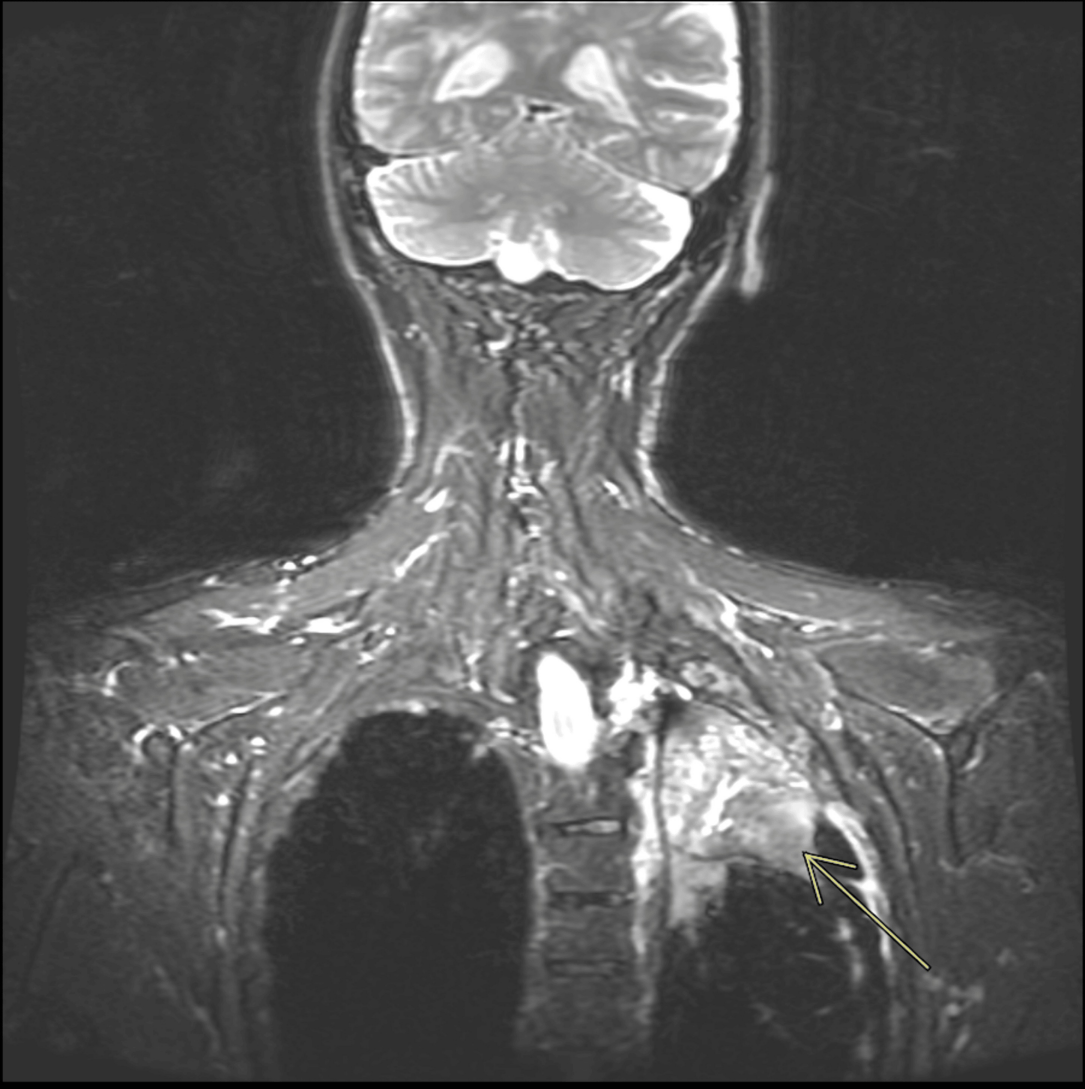
Coronal T2 MRI sequence with fat suppression of the brachial plexus showing possible impingement of the left recurrent laryngeal nerve. No definite evidence of brachial plexus involvement The arrow points to a heterogeneously enhancing soft tissue mass at the left lung apex measuring 57 x 87 x 87 mm

Unfortunately, by late 2012, the patient eventually developed new-onset pain in his left chest, mild dyspnea, dysphagia, hoarseness, easy fatigability, and 7 pounds of unintentional weight loss. Physical examination revealed left Horner’s syndrome and left shoulder pain. CT thorax and abdomen revealed a new mass in the LUL of his lung suggesting recurrence of the Pancoast tumor and no change in the oncocytoma (Figure 8). A later brachial plexus MRI showed impingement of the left recurrent laryngeal nerve, which explained his loss of voice but no evidence of brachial plexus involvement (Figure 9). The left intercostal nerves 2-6 seemed to be affected on the MRI, which correlated with the clinical finding of tingling and paresthesias in his left upper chest.

**Figure 10:**
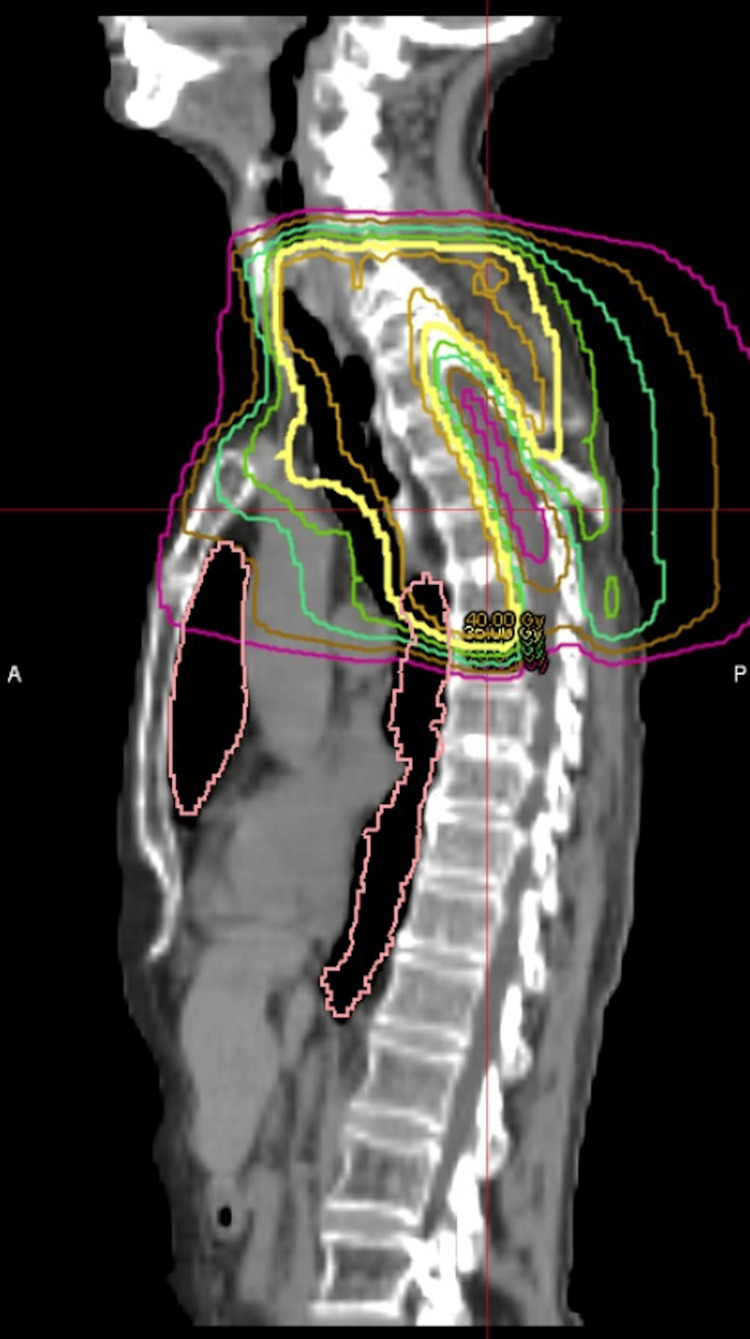
Palliative reirradiation plan targeting the recurrent Pancoast tumor of the left lung Sagittal CT view. No GTV is shown. GTV: gross tumor volume

**Figure 11:**
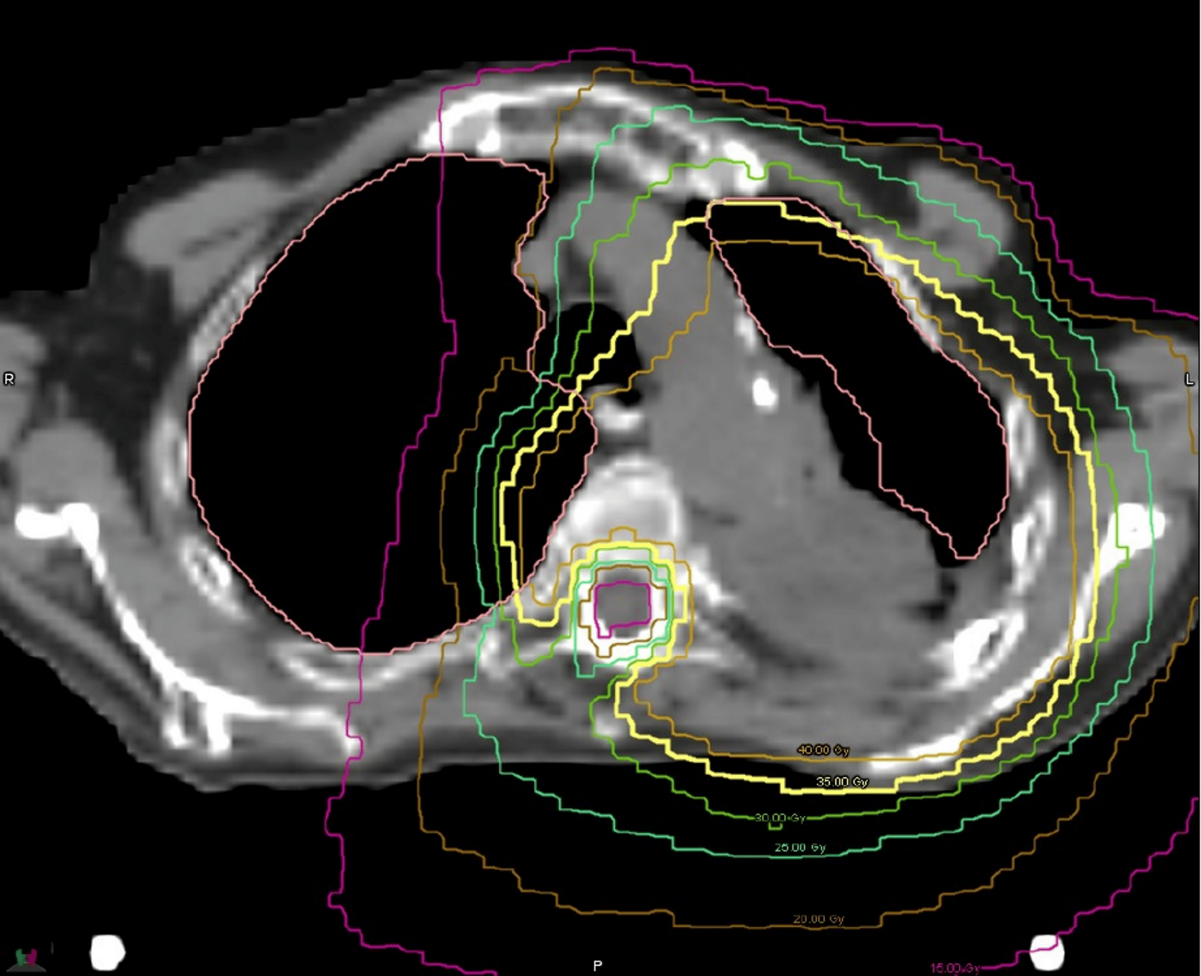
Palliative reirradiation plan targeting the recurrent Pancoast tumor of the left lung Axial CT view. No GTV is shown. GTV: gross tumor volume

**Figure 12:**
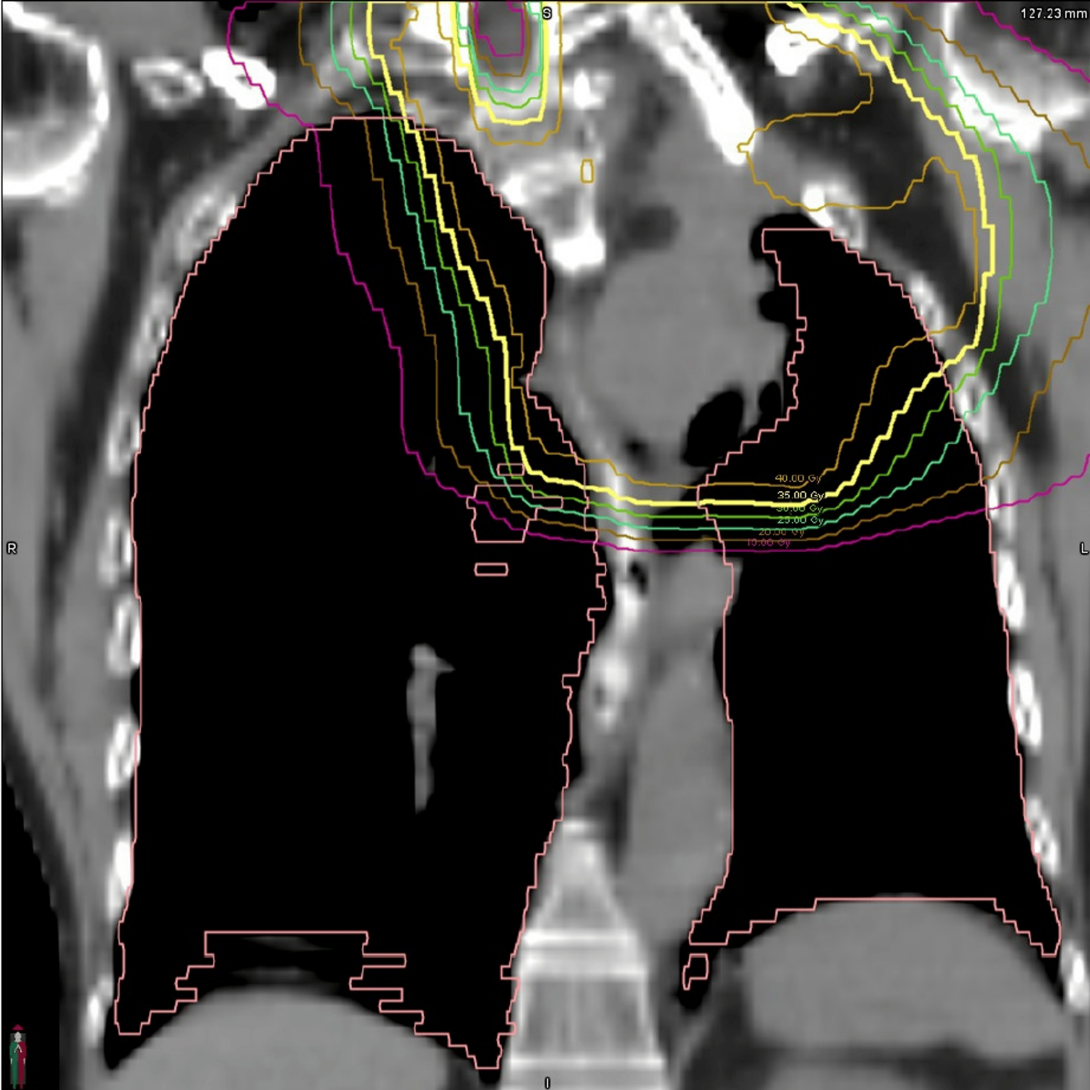
Palliative reirradiation plan targeting the recurrent Pancoast tumor of the left lung Coronal CT view. No GTV is shown. GTV: gross tumor volume

A biopsy was not performed because the patient did not want to delay treatment but rather begin palliative therapy as soon as possible. The patient declined chemotherapy but was treated with palliative radiation therapy in March-April 2013, specifically precision tomotherapy of 40 Gy in 20 fractions (Table 4), which mitigated the risk of acute treatment side effects such as esophagitis. Radiation therapy targeted the left supraclavicular, brachial plexus, and LUL areas (Figures 10-12).

**Table 4:** Radiation dose-volume constraints for various pertinent regions of interest (ROIs) for palliative reirradiation of the recurrent left Pancoast tumor PRV: planning risk volume; CTV: clinical target volume; IGTV: internal gross tumor volume; EVAL: evaluation; PTV: planning target volume

Region of interest	Maximum dose (Gy)	Mean dose (Gy)
CORD	27.95	8.2
CORD PRV NEW	42.75	12.24
CTV 0% and 50% phase (IGTV)	44.62	41.41
CTV40	44.62	41.02
CTV40EVAL	44.62	41.35
Esophagus	43.06	23.49
Heart	20.76	1.57
Left brachial plexus	35.14	33.56
Left lung	42.85	11.95
Lung EVAL	44.44	8.09
Brachial plexus inside target	34.33	33.96
Brachial plexus margin	35.25	34.11
PTV40	45.66	40.85
PTV40EVAL	45.66	41.32
Right lung	44.44	6.24

This radiation therapy was well tolerated by the patient. He had no pain or neurological side effects, with the only adverse effect being mild hyperpigmentation (NCI CTCAE Grade 1) of the skin on his upper back. The patient also saw otolaryngology for left vocal cord augmentation with Radiesse due to his poor quality of voice and occasional choking. This procedure significantly improved his phonatory function, and he was quite pleased with the vocal result (which also helped prevent future aspiration events). Overall, the patient was very grateful for these palliative treatments and described them as giving him a good quality of life and reducing his pain from severe to a self-reported 1/10, and he had no need for any pain medication.

**Figure 13:**
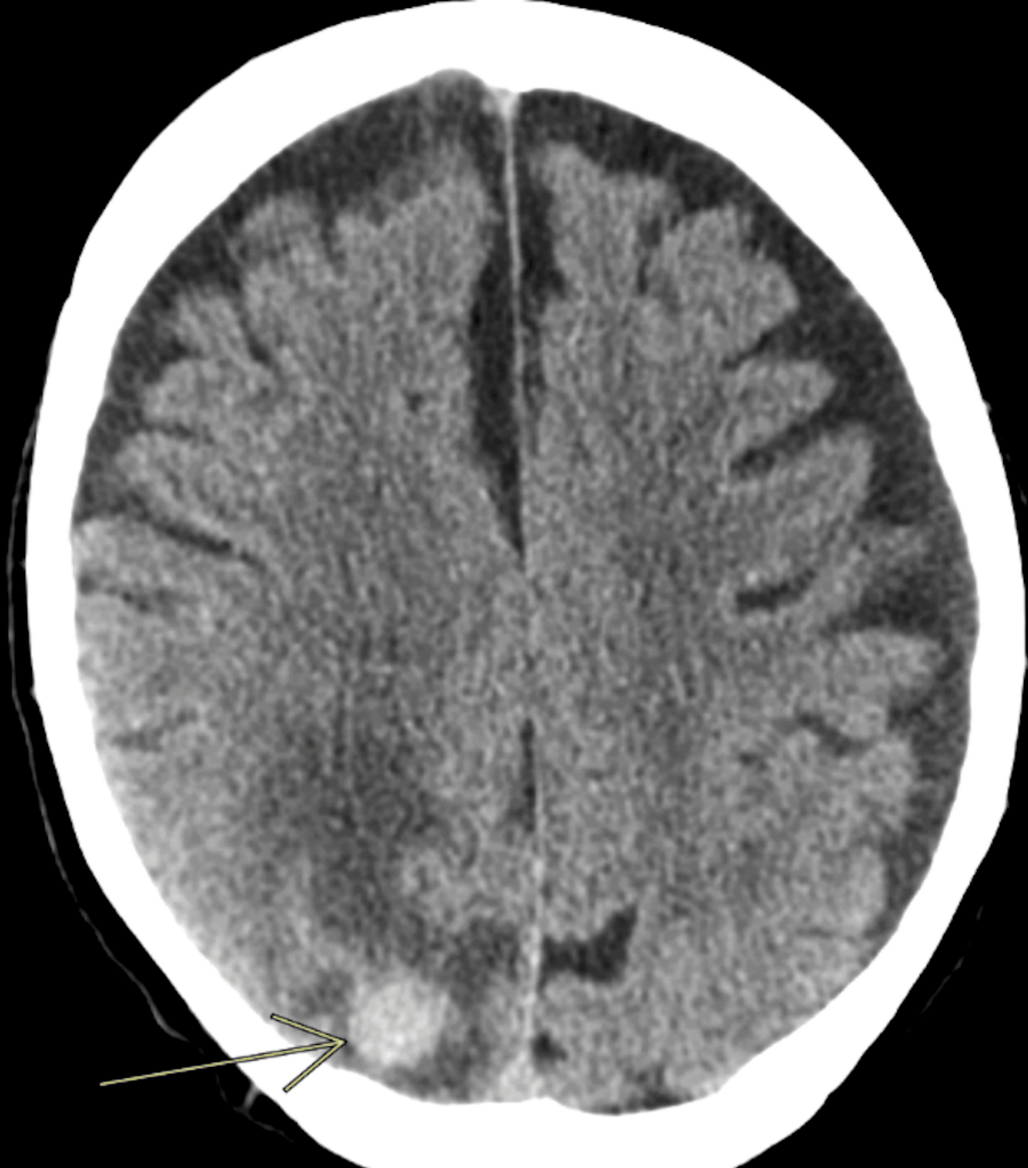
CT of the head/brain window revealing a rounded intra-axial 14 mm hyperdense right occipital lobe mass, presumed to be a metastasis from his Pancoast tumor with a moderate amount of perilesional white matter hypodensity/vasogenic edema The arrow points to the occipital lobe lesion

He did well for roughly 10 months after these palliative measures, but unfortunately developed chest pain, dyspnea, and significant unintentional weight loss (30 pounds in four months) in late 2013. He then had a fall in late January of 2014, and a CT of his head revealed a 14 mm right occipital lobe mass, presumed to be a metastasis from his lung cancer (Figure 13).

Treatment for this was decided to be simply surveillance. He was discharged home but continued to decline functionally until the progression of his chest pain and increasing weakness prompted a return to the emergency room. The patient pursued comfort care in the hospital with respect to pain and symptom management, which involved 0.5 mg hydromorphone prescribed for pain and dyspnea. He continued to decline before becoming comatose and subsequently dying in the hospital, with the cause of death attributed to metastatic lung cancer to the brain. No autopsy was performed.

## Discussion

Treatment of renal oncocytoma

Renal oncocytomas make up about 18% of SRMs and most commonly present between the sixth and eighth decades of life. They are more common in men with a male-to-female ratio ranging from 1.25:1 to 3.3:1 [10]. Differentiating an oncocytoma from other renal lesions is crucial since management and prognosis vary depending on the precise pathology. Excluding RCC, especially chromophobe RCC (ChRCC), is of paramount importance given its malignant potential and radiologic and pathologic similarities to an oncocytoma.

Because over 75% of patients with a renal oncocytoma are asymptomatic, the first step in work-up often is CT imaging done for another indication. On CT, a renal oncocytoma appears as an iso- or hypodense solid mass in the renal cortex and enhances with contrast, but these features are non-specific and are also observed in RCC, among other lesions [11]. Thus, given the shortcomings of imaging alone, a biopsy is often pursued. The only circumstances where a biopsy is avoided are in patients who request removal of the lesion even if benign and in palliative patients who will be managed conservatively regardless of the biopsy results [12]. In our case, a biopsy was important for several reasons: the imaging could not show definitively if the lesion was a benign renal oncocytoma, the patient was not palliative, and metastasis to the kidney from his previous Pancoast tumor was a possibility (such metastasis is rare but has been documented as approximately 1% of NSCLC metastases in one study) [13].

Histologically, renal oncocytomas broadly show a solid/nested pattern of growth with eosinophilic cytoplasm. This pattern of growth resembles various entities, most closely ChRCC. The pathologic differential diagnosis thus includes ChRCC, succinate dehydrogenase (SDH)-deficient RCC, and angiomyolipoma among other lesions [3]. The main diagnostic dilemma is between oncocytoma and ChRCC, but unlike the latter, oncocytomas generally have fewer nuclear membrane irregularities and a far denser vascular network, whereas ChRCC shows more trabecular growth. Cytokeratin 7 is usually negative in oncocytoma but may be focally positive in clear cell RCC and is positive in papillary and chromophobe RCC. Colloidal iron staining can also distinguish oncocytoma from RCC in that the former typically has negative or luminal staining whereas the latter has reticular cytoplasmic staining [3].

Treatment for SRMs, including oncocytomas, broadly falls into three categories: surgery, thermal ablation, and active surveillance. Surgery is typically nephron-sparing (i.e., partial nephrectomy) and is preferred for lesions with characteristics more concerning for malignancy including larger size, complex architecture, and some biopsy findings if applicable. When the SRM is sufficiently small (<3 cm), as in our case, ablative procedures such as cryotherapy or radiofrequency ablation are viable options if the patient cannot tolerate surgery or decides against it [12]. However, these treatments were not options for our patient at the time. Indeed, the American Urological Association only approved ablative procedures as viable alternatives to nephron-sparing surgery for SRMs in 2017 [5]. Finally, for lesions with the lowest risk of malignant potential (e.g., size <2 cm, cystic features), active surveillance is now considered a viable option, although it was not the standard of care at the time of this case. Studies have found low rates of growth (0.1 cm/year) and extremely low rates of metastasis in patients undergoing active surveillance for appropriately selected SRMs [14]. Nevertheless, even SRMs suggestive of benign lesions like oncocytomas are often treated as they can cause symptoms including hematuria and flank pain and rarely can metastasize [4]. In our case, the patient's lesion was discovered incidentally and he was originally asymptomatic but it was a possibility that this lesion could become symptomatic in the future. Also, treatment was considered important given the possibility of metastasis from the Pancoast primary and the lack of a definitive diagnosis of oncocytoma from pathology, as previously mentioned. Moreover, active surveillance was not considered a viable management option at the time of this case [14].

Radiation has long been used to treat kidney lesions, but only for RCC metastasis or recurrence, not primary tumors. Indeed, until very recently, surgery was the only therapeutic intervention for primary RCC after research in the late 1990s showed this cancer was relatively radioresistant [11]. Thus, either radical or partial nephrectomy combined with palliative radiotherapy (if deemed appropriate later on) was the standard of care until the advent of SBRT. SBRT fundamentally changed the role of radiotherapy in kidney tumors and RCC in particular due to its ability to provide high-dose radiation, often upwards of 20 Gy per fraction, compared to less than 2-3 Gy with conventional radiotherapy. This drastic escalation of dose made SBRT an option for not only recurrence and metastatic disease but also primary tumors. Currently, surgery remains the first line treatment for resectable RCC, but SBRT has shown efficacy as a primary treatment and is preferred in some scenarios such as single functional kidney and bilateral renal tumors [11]. Thus, SBRT is a viable treatment option for RCC in general; however, to the best of our knowledge, we report one of the first documented clinical uses of SBRT for the treatment of renal oncocytoma. Of note, a recent study examined the effect of SBRT on a range of renal tumors, including oncocytoma, and found this therapy to provide excellent control of these lesions, thus essentially confirming our results with a larger sample size [6].

Therefore, in the context of an expanding role for radiotherapy in the treatment of kidney lesions, we document one of the first cases of its use for an oncocytoma. Surgery, and in recent years, an ablative procedure, is often sufficient for the treatment of SRMs. However, particularly in cases where the diagnosis is not certain (as in our case) radiotherapy could serve a role in treatment, as it covers the potential for a more sinister diagnosis such as RCC. Our case is a real-world example of this dynamic; SBRT successively maintained our patient’s quality of life and prevented symptom development and progression (and potentially metastasis) of the lesion.

Treatment of Pancoast tumor

In recent decades, the treatment of Pancoast tumors has radically improved due to better-quality radiation therapy, improved surgical techniques, and new chemotherapy drugs. Induction neoadjuvant chemoradiation followed by surgery (which can be technically challenging due to tumor location issues) has emerged as the standard of care for Pancoast tumors. Platinum-based chemotherapy agents (cisplatin, carboplatin, etc.) combined with etoposide along with concurrent thoracic radiotherapy is the typical neoadjuvant chemoradiation regimen [9]. Surgical management may include removal of the upper lobe of the lung, subclavian artery/vein, ribs, vertebral bodies, branches of the brachial plexus, and mediastinal lymphadenectomy. The neoadjuvant chemoradiation-surgery tri-modality treatment technique has been shown to provide 5- and 10-year disease-free survival rates of 47.1% and 28.2% respectively, and 5-, and 10-year overall survival rates of 50.1% and 31.8%, respectively [15].

Our case is an example of the success of treating a patient with a novel oncology treatment regimen that was first initiated at the Verspeeten Family Cancer Centre (formerly the London Regional Cancer Program) in 1996. Known as the VCRT (vinblastine, cisplatin, and radiation therapy) regimen, it has emerged as the standard curative-intent treatment protocol for unresectable stage III NSCLC patients at the Verspeeten Family Cancer Centre. This protocol consists of two cycles (each three days duration) of vinblastine and cisplatin at three-week intervals followed by a subsequent third and fourth cycle (again, three days duration) of vinblastine and cisplatin administered concurrently with radiation therapy (60 Gy over 30 fractions) (Table 1). The chemotherapy agents vinblastine and cisplatin help control micro-metastatic disease and cisplatin acts additionally as a radio-sensitizer. Vinblastine dose reduction during concurrent radiation therapy is designed to prevent toxic side effects such as esophagitis (which was successfully avoided in our patient). A retrospective analysis of patients with unresectable stage III NSCLC treated with the VCRT protocol at Verspeeten Family Cancer Centre between 1996 and 2006 revealed a median survival time of 18.2 months and a five-year overall survival duration of 19.8% [16]. Our patient tolerated the VCRT protocol with minimal side effects that all resolved (pancytopenia with no fevers and peripheral neuropathy). It also caused resolution of his chest and arm pain and had an overall excellent result of giving the patient nine years disease-free with no symptoms. Overall, our case demonstrates the effectiveness of using the VCRT protocol for surgically non-resectable NSCLC Pancoast tumors, and clinicians should consider using it in patients who meet this description [16,17].

Lastly, this case is an example of successful palliative reirradiation for local recurrent lung cancer after previous successful radiation therapy. Retreatment after previous chemoradiation can be very difficult due to cancer cells often showing resistance to further treatment and reirradiation causing further radiation injury (such as radiation pneumonitis) [18]. Hence, reirradiation of locally recurrent lung cancer is rarely indicated [19]. Also, it is still relatively unknown whether any possible survival benefits of high-dose re-irradiation outweigh exposing the patient to further toxicity, or whether palliative radiation therapy (a lower dose) should be utilized instead. In our case, precision tomotherapy of 40 Gy in 20 fractions was used to palliatively re-radiate our patient when his Pancoast tumor recurred after living nine years disease-free. This was very effective in palliation: it controlled his symptoms, improved his quality of life for 10 months (median survival is normally only five months in palliative reirradiation of NSCLC), and prevented progression to brachial plexus or spinal cord damage [18].

## Conclusions

While oncocytomas are common benign lesions of the kidney, they can still present diagnostic challenges and sometimes bothersome symptoms such as hematuria and flank pain. In patients where the pathologic diagnosis is uncertain or the standard treatments of surgical excision and ablation are either contraindicated or declined, our case describes how SBRT can be a successful management option. This unique case also demonstrates that the VCRT protocol used for unresectable NSCLC tumors can be used successfully for a Pancoast tumor and in our case, provide an excellent result of nine years of disease-free survival. Lastly, this case shows that palliative re-irradiation of a recurrent Pancoast tumor can be employed to provide successful symptom management, control progression, and provide many months of survival without exposing a patient to toxic side effects that might occur in high-dose re-irradiation.

## References

[1] Williams GM, Lynch DT (2025). Renal oncocytoma. StatPearls [Internet].

[2] Choudhary S, Rajesh A, Mayer NJ, Mulcahy KA, Haroon A (2009). Renal oncocytoma: CT features cannot reliably distinguish oncocytoma from other renal neoplasms. Clin Radiol.

[3] Wobker SE, Williamson SR (2017). Modern pathologic diagnosis of renal oncocytoma. J Kidney Cancer VHL.

[4] Perez-Ordonez B, Hamed G, Campbell S, Erlandson RA, Russo P, Gaudin PB, Reuter VE (1997). Renal oncocytoma: a clinicopathologic study of 70 cases. Am J Surg Pathol.

[5] Bertolotti L, Bazzocchi MV, Iemma E (2023). Radiofrequency ablation, cryoablation, and microwave ablation for the treatment of small renal masses: efficacy and complications. Diagnostics (Basel).

[6] Sun MR, Brook A, Powell MF, Kaliannan K, Wagner AA, Kaplan ID, Pedrosa I (2016). Effect of stereotactic body radiotherapy on the growth kinetics and enhancement pattern of primary renal tumors. AJR Am J Roentgenol.

[7] Francis R, Goldstein AL, Akar FA (2024). Pancoast tumors. Video-assist Thorac Surg.

[8] Elsaka O, Noureldean MA, Gamil MA, Ghazali MT, Abd Al-Razik AH, Hisham D (2022). Pathophysiology, investigations, and management in cases of Pancoast tumor.

[9] Lin TY, Atrchian S, Humer M, Siever J, Lin A (2021). Clinical outcomes of pancoast tumors treated with trimodality therapy. J Thorac Dis.

[10] Trevisani F, Floris M, Minnei R, Cinque A (2022). Renal oncocytoma: The diagnostic challenge to unmask the double of renal cancer. Int J Mol Sci.

[11] Mirkheshti N, Farrukh N, Legesse T, Rowe SP, Gordetsky J, Hussain A (2022). Renal oncocytoma: a challenging diagnosis. Curr Opin Oncol.

[12] Campbell SC, Uzzo RG, Karam JA, Chang SS, Clark PE, Souter L (2021). Renal mass and localized renal cancer: evaluation, management, and follow-up: AUA guideline: part II. J Urol.

[13] Niu FY, Zhou Q, Yang JJ (2016). Distribution and prognosis of uncommon metastases from non-small cell lung cancer. BMC Cancer.

[14] McIntosh AG, Ristau BT, Ruth K (2018). Active surveillance for localized renal masses: tumor growth, delayed intervention rates, and >5-yr clinical outcomes. Eur Urol.

[15] Hutchings HE, Cox J, Westra J, Kuo YF, Okereke IC (2023). Treatment patterns and outcomes in patients with Pancoast tumors: a national cancer database analysis. J Thorac Dis.

[16] Waters E, Dingle B, Rodrigues G (2010). Analysis of a novel protocol of combined induction chemotherapy and concurrent chemoradiation in unresected non-small-cell lung cancer: a ten-year experience with vinblastine, cisplatin, and radiation therapy. Clin Lung Cancer.

[17] Sause W, Kolesar P, Taylor S IV (2000). Final results of phase III trial in regionally advanced unresectable non-small cell lung cancer: Radiation Therapy Oncology Group, Eastern Cooperative Oncology Group, and Southwest Oncology Group. Chest.

[18] De Ruysscher D, Faivre-Finn C, Le Pechoux C, Peeters S, Belderbos J (2014). High-dose re-irradiation following radical radiotherapy for non-small-cell lung cancer. Lancet Oncol.

[19] Grambozov B, Nussdorfer E, Kaiser J (2021). Re-irradiation for locally recurrent lung cancer: a single center retrospective analysis. Curr Oncol.

